# Notre expérience de méningiome intracrânien à Dakar: à propos de 50 cas

**DOI:** 10.11604/pamj.2015.20.379.6070

**Published:** 2015-04-16

**Authors:** Alioune Badara Thiam, Yannick Canton Kessely, Mbaye Thioub, Maguette Mbaye, Mouhamed Faye, El Hadj Cheikh Ndiaye Sy, Ndaraw Ndoye, Momar Codé Ba, Youssoupha Sakho, Seydou Boubacar Badiane

**Affiliations:** 1 Service de Neurochirurgie du Centre Hospitalo-universitaire de Fann, Dakar, Sénégal

**Keywords:** Méningiome, chirurgie, tumeur bénigne, Meningioma, surgery, benign tumor

## Abstract

Les méningiomes sont des tumeurs bénignes extra parenchymateuses développées aux dépens des villosités arachnoïdiennes. Ils tirent leur gravité de leur localisation dans des zones hyperfonctionnelles. Le but de cette étude est d’évaluer la prise en charge des méningiomes intracrâniens depuis la réhabilitation du service de neurochirurgie en 2007. Les auteurs rapportent une série rétrospective portant sur 50 patients admis et opérés d'un méningiome intracrânien d'octobre 2007 à Juin 2013 dans leur service. Ont été inclus les patients ayant un dossier complet. Les patients étaient âgés de 08 à 70 ans avec une moyenne d’âge de 47,3 ans, 68% avaient plus de 40 ans. La sex-ratio était de 0,76. Les manifestations cliniques étaient essentiellement l'hypertension intracrânienne (46%), le déficit moteur (30%) et la comitialité (48%). La tomodensitométrie cérébrale était réalisée chez 41 patients et l'IRM chez 23. Le méningiome était localisé sur la convexité chez 24 patients. L'imagerie postopératoire immédiate était réalisée chez 15 patients. L'exérèse a consisté dans 44% des cas à un Sympson II, et dans 30% des cas à un Sympson III. L'anatomopathologie a conclu à une prédominance du type méningothélial (38%) et 60% des méningiomes étaient de grade I selon la classification de l'O.M.S. Aucun patient n'a bénéficié d'une radiothérapie. La mortalité était de 16%. Les pays d'Afrique subsaharienne continuent d'accuser un retard malgré les efforts réalisés dans les domaines diagnostic et thérapeutique des méningiomes intracrâniens. Le pronostic s'est considérablement amélioré à mesure de l'amélioration du plateau technique dans notre pays.

## Introduction

Les méningiomes sont des tumeurs bénignes extra parenchymateuses développées aux dépens des villosités arachnoïdiennes. Ils tirent leur gravité de leur localisation dans des zones hyperfonctionnelles. Après la réhabilitation du service en 2007 et suite à la publication de 1999 [1] portant sur les méningiomes, l’équipe de neurochirurgie 2007 par cette étude décide d’évaluer la prise en charge des méningiomes intracrâniens au CHU de Fann.

## Méthodes

Il s'agit d'une série rétrospective (octobre 2007 à Juin 2013) portant sur 50 patients opérés d'un méningiome intracrânien. Ont été inclus dans cette série les patients ayant bénéficié d'une imagerie cérébrale et dont le dossier comportait, un rapport opératoire précisant la qualité de l'exérèse, une étude histologique et un suivi post opératoire sur une période d'au moins un an.

## Résultats

**Figure 1:**
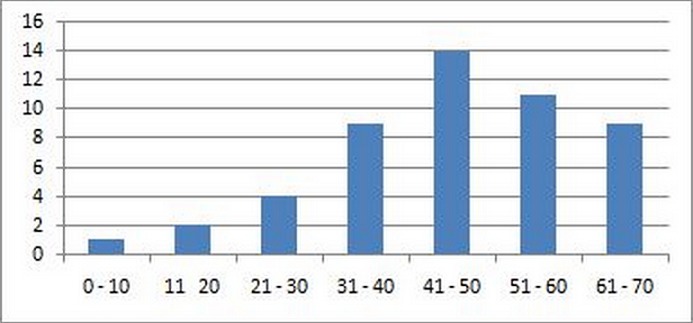
Répartition des malades selon l’âge

Sur un total de 78 patients suivis et opérés de méningiome intracrânien durant la mémé période, 50 ont satisfait aux critères de cette étude. Ainsi les 50 patients concernés étaient âgés de 08 à 70 ans avec une moyenne d’âge de 47,3 ans parmi lesquels on comptait 28 femmes, soit 56% (Figure 1). Le Sex-ratio était de 0,76. Les patients âgés de plus de 40 ans étaient au nombre de 34soit 68% des cas et un seul patient avait moins de 10 ans. Sur les 28 femmes, 23(82%) étaient âgées de plus de 40 ans et 18 (64%) de plus de 45 ans. La répartition selon l’âge des patients est présentée dans la Figure 1. Dans les antécédents, l'hypertension artérielle a été retrouvée chez 4 patients. Deux patients étaient diabétiques de type II sous insulinothérapie, et deux autres avaient été opérés de tumeur cérébraledont le diagnostic de méningiome avait été évoqué à l'imagerie, mais la nature histologique non précisée. Une patiente a été opérée pour sinusite maxillaire en oto-rhino-laryngologie. Le début de la symptomatologie était progressif chez 60% des patients par l'installation des céphalées au long cours, et rebelles au traitement. Le délai moyen avant l'hospitalisation était de 6 mois avec des extrêmes de 15j à 3 ans. Sur le plan clinique, les manifestations étaient essentiellement représentées par les signes oculaires: exophtalmie chez 7 patients et une cécité bilatérale chez 3autres. Une anosmie a été observée chez 3 patients et une hypoacousie unilatérale a été décelée dans un cas. Une comitialité a été présente chez 29 patients. Vingt patients avaient présenté une hémiparésie dont 10 à gauche, et trois, une hémiplégie. Un syndrome cérébelleux cinétique a été objectivé dans un cas ainsi qu'une paraparésie. La moitié des patients n'avait aucun déficit. Les troubles de langage étaient présents chez 8 patients: une aphasie de Broca chez 3 patients, et une dysarthrie chez les 5 autres. Une hypertension intracrânienne clinique (céphalées, vomissements, troubles de la vision) était présente dans 21 cas. Le processus expansif intracrânien a été découvert de façon fortuite chez 2 patients au cours d'une imagerie faite pour un traumatisme crânio-encéphalique, chez une patiente âgée de 08 ans, et chez une patiente référée par le service d'ORL après réalisation d'une tomodensitométrie cérébrale dans le cadre d'investigation d'une sinusite.

**Figure 2:**
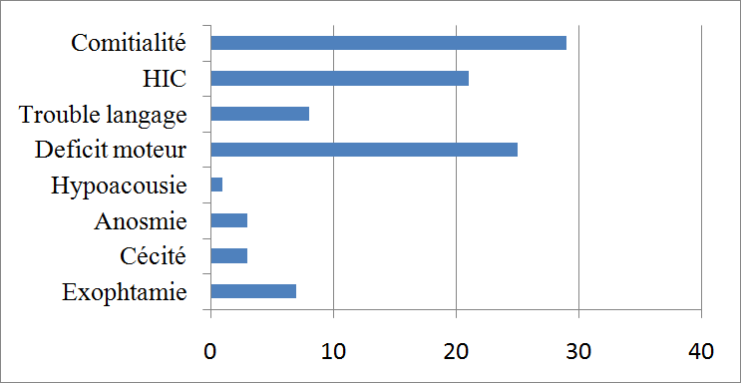
Présentation clinique

Les troubles de l'humeur chez 3 patients, ainsi qu'une altération de la fonction cognitive chez un patient ont motivé une consultation en psychiatrie. Le diagnostic a été fait en période de post-partum chez une patiente. Un déficit moteur isolé a été le motif de consultation chez 7 patients (Figure 2). Un électroencéphalogramme a été réalisé chez 24 patients et 22 étaient en faveur d'une souffrance corticale diffuse, un pour une épilepsie partielle, et un normal. Tous les patients ont bénéficié d'une imagerie cérébrale mettant en évidence un processus expansif intracrânien. Une tomodensitométrie cérébrale préopératoire avec et sans injection de produit de contraste a été réalisée chez 41 patients, parmi ceux-ci 14 ont réalisé en sus, une imagerie par résonnance magnétique (IRM). Neuf patients avaient réalisé d'emblée une IRM. Le Tableau 1 représente la répartition selon la localisation. Une méningiomatose (association dans un cas, d'un méningiome de topographie sphéno-orbitaire et d'un méningiome fronto-pariétal, et dans le second cas, d'un méningiome sphéno-orbitaire et d'un méningiome pariétal) a été décrite notamment chez 2 patients présentant neurofibromatose II. Aucune localisation ventriculaire n'a été observée. Différentes autres lésions ont été décrites à l'imagerie. Il s'agissait d'un œdème périlésionnel chez 3 patients, d'un effet de masse chez 4 et d'un envahissement du sinus sagittal supérieur chez 4 autres. Par ailleurs l'envahissement du torcular, la compression de l'artère sylvienne, la nécrose intra-tumorale et le kyste intratumoral ont été rapporté chacun chez un patient. Cinq patents ont présenté des calcifications intratumoraux, 3 une extension extracrânienne dont deux en pariétale et une en fronto-pariétale, et 2 une hyperostose dont une sphéno-temporale et une pariétale. Le signe de la queue de la comète a été présent chez 17 patients.

**Tableau 1:** Répartition topographique

**Convexité**	**24**	Pariétal	10		
		Fronto-pariétal	6		
		Pariéto-occipital	3		
		Temporal	3		
		Fronto-temporal	2		
**Base**	**16**	Ethmoïde (lame criblée)	4		
		Sphénoïde	12	*Jugum*	3
				*Petite aile*	4
				*Grande aile*	2
				*Séllaire*	1
				*Sphéno orbitaire*	2
**Fosse postérieure**	**6**	Clivus	4		
		Torcular	1		
		Ecaille occipitale	1		
**Faux**	**1**				
**Parasagittal**	**3**				

**Figure 3:**
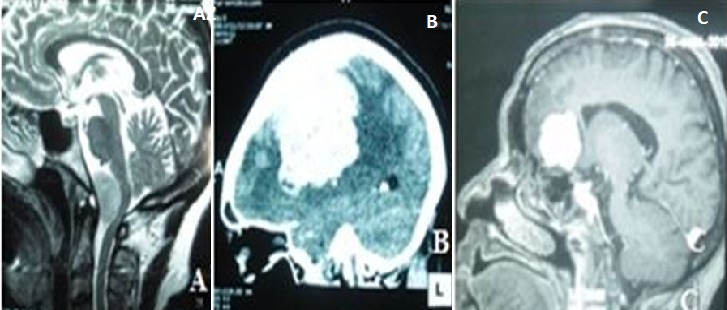
A) IRM en T1 reconstruction sagittale mettant en evidence un méningiome du clivus avec un épaississement dural; B) TDM cérébrale, réconstruction sagittale montrant un volumineux processus intracranien de la convexité se rehaussant au produit de contraste avec une réaction osseuse; C) IRM montrant un processus expansif olfactif se rehaussant fortement au produit de contraste

Quinze patients ont pu réaliser une tomodensitométrie post-opératoire mettant en évidence un résidu tumoral chez 3 patients, une exérèsecomplète dans 6 cas, un œdème cérébral dans 3 cas, un hématome du foyer opératoire chez 2 patients, une cavité postopératoire chez un patient. Aucune IRM n'a été faite après l'intervention chirurgicale (Figure 3). Le traitement médical était à base d'antalgiques, d'anticonvulsivants et d'anti-œdémateux. Les patients ont bénéficié d'un traitement chirurgical qui a consisté en la réalisation d'un volet et d'une exérèse tumorale par morcellement. La qualité de l'exérèse était appréciée par le grading de Simpson, ainsi il y avait eu 9 (18%) du grade I, 22 (44%) du grade II, 15 (30%) du grade III et 4 (8%) du grade V. Les types histologiques rapportés étaient: méningothelial (40%), atypique (22%), transitionnel 10%, fibroblastique 10%, psammomateux 8%, choroide 4% et angiomateux 6% Trente patients soit 60% étaient porteurs de méningiome de grade I selon la classification de l'O.M.S, 15 (30%) de grade II et 5(10%) de grade III. Aucun patient n'a bénéficié d'une radiothérapie. La mortalité post-opératoire a concerné 8 patients (Tableau 2) dont 7 étaient âgés de plus de 50 ans parmi lesquels 5 hommes. Un patient est décédé sur table opératoire par trouble hémodynamique. Les trois patients chez qui l'imagerie avait mis en évidence une lyse osseuse (2 de topographie pariétale, et une pariéto-temporale) avec une extension extra crânienne de la tumeur sont tous décédés. Pour les lésions de topographie pariétale et pariéto-frontale, les décès étaient imputés à une pneumopathie chez 2 patients, des troubles respiratoires chez un patient et à un œdème aigu du poumon dans un cas. Dans l’évolution, six patients ont été perdus de vue. Une récidive tumorale a été constatée à l'imagerie de contrôle au 12^ème^ mois. Les trois patients aveugles (cécité bilatérale) avaient un état clinique stationnaire malgré le traitement chirurgical. Après la première année de suivi, 8 autres patients ont été perdus de vue. Les 19 autres ont repris leurs activités. La morbidité est rapportée dans des proportions différentes et essentiellement faite de convulsions postopératoire (chez 2 patients), d'anosmie bilatérale (chez 2 patients), d'un cas de syndrome infectieux, de méningocèle postopératoire, de suppuration de la plaie, de fuite du liquide cérébro-spinal et de collection sous-durale. Dans 3 cas le tableau est resté inchangé et 3 patients ont présenté un œdème cérébral.

**Tableau 2:** Répartition de décès en fonction de la topographie, du type histologique et de la qualité de l'exérèse chirurgicale

Topographie		Décès	Histologie	Sympson
Pariétale	10	2	Psammomateux	II (2)
			Atypique	
Pariéto-frontale	6	2	Méningothélial	II ; III
			Transitionnel	
Clivus	4	2	Fibroblastique	IV (2)
Jugum sphénoïdal	3	1	Atypique	III
Petite aile du sphénoïde	4	1	Méningothélial	III

## Discussion

Le méningiome a fait l'objet de nombreuses études et à l'heure actuelle les recherches sont le plus, orientées dans le domaine de la microbiologie et l'oncogenèse de cette pathologie [2, 3]. La prévalence confirmée de la pathologie est estimée à 97,5/100000 aux Etats Unis avec plus de 170. 000 patients réellement diagnostiqués vivant avec cette pathologie [4]. La prévalence de cette pathologie en Afrique subsaharienne est souvent difficile à établir avec précision pour plusieurs raisons: l'insuffisance de couverture sanitaire efficace en matière d'infrastructure, l'absence d'imagerie médicale, l'insuffisance des ressources humaines et le manque de registre de tumeurs. Ainsi, bon nombre de cas n'accèdent pas, ou tardivement aux structures de santé. Tous les méningiomes n'ont pas été traités chirurgicalement, les méningiomes infra-cliniques diagnostiqués sur les séries autoptiques sont rares alors qu'ils relèvent de 1,4% [5]. En 2008, en Côte d'Ivoire Ndri et coll [6] sur une période de 10 ans ont pris en charge 114 patients porteurs de méningiome dont 96 documentés. Au Sénégal, sur une période de 3 ans et demi, Sakho et coll en 2005 [7] ont opérés 55 patients porteurs de méningiome dont 24 dossiers complets. Nous avons constaté sur une période de notre étude (5 ans et 8 mois),78 patients porteurs de méningiome intracrâniens ont été pris en charge sur un total de 428 tumeurs cérébrales opérés durant la même période, soit une fréquence de 18,22%. Ibebuike et coll [8] en Afrique du sud ont rapporté une incidence du méningiome à 31,8%, Wilfred et coll [9] au Nigeria, ont relevé au cours au cours de leur étude une incidence de 23,8%. Pour le CBTRUS (Central Brain Tumour Registry of the United States) [10] aux Etats Unis, les méningiomes ont représenté de 2004 à 2006 34% des tumeurs cérébrales primitives et surviennent avec une fréquence de 12.8/1.00.000. Une étude épidémiologique américaine est arrivée à la conclusion que les méningiomes sont plus fréquents chez les sujets noirs que chez les hispaniques et caucasiens [4]. Nous n'avons qu'une série composée de sujets noirs, et pensons que les investigations génétiques apporteront plus de clarifications à ce propos. Contrairement à Badiane et coll [1] en 1999 à Dakar qui avaient montré que le méningiome est une tumeur de la cinquantaine, 28% de nos patients étaient dans la tranche 40-50 ans et 22% dans celle 50-60 ans. Conformément à la littérature [11], le sex-ratio lors de notre étude est en faveur des femmes ce qui s'expliquerait par la présence des récepteurs hormonaux. Selon Claus et coll [2], l'incidence croissante des méningiomes durant le pic de reproductivité femme/homme est de 3,15:1 et de 2:1 en période post-pubertaire ce qui correspond à nos données. Le méningiome est exceptionnel chez l'enfant (6%), deux de nos patient étaient âgés respectivement de 08 et 11 ans. Ceci corrobore la conclusion de l’étude de Nikki et coll [12] portant sur le méningiome intracrânien en milieu pédiatrique à propos de 72 cas sur une période de 36 ans concernant la rareté du méningiome dans cette tranche d’âge et le caractère beaucoup plus agressif que celui de l'adulte. Celui de notre jeune patiente ne l’était pas. Claus [2] a rapporté lors de son étude que le traumatisme crânien a été considéré depuis le temps de Harvey Cushing comme un «facteur de risque» de méningiome. Dans leur série, Eskandary et coll [13] ne rapportent pas de telles constatations.

Deux fois dans notre série, le méningiome a été découvert lors d'un traumatisme crânien qui a nécessité la réalisation d'une tomodensitométrie cérébrale. Aucun facteur étiologique n'a été décelé dans notre étude. Le facteur de risque environnemental le plus important, identifié pour la survenue du méningiome est l'exposition à l'irradiation ionisante [14]. Selon Claus et coll [14], l'exposition à l'irradiation de la radiographie panoramique dentaire réalisée dans un passé récent quand la dose était élevée, semble être associé à un risque élevé de méningiome intracrânien, surtout si cette radiographie a été réalisé chez le patient avant l’âge de 10 ans. Aucun de nos patients n'en a eu recours. La radiothérapie pour tumeur cérébrale comme facteur étiologique des méningiomes [15] n'a non plus pas été retrouvée. D'après Umansky et coll [16] le seul facteur de risque exogène approuvé dans la survenue des méningiomes est l'irradiation, et aucun de nos patients n'en a bénéficié. La convexité est le siège de prédilection de la lésion au cours de notre étude comme l'ont rapporté Stephane et coll [17] ainsi que certaines séries africaines [1, 6, 7]. Wilfred et coll [9] par contre au Nigeria ont rapporté une prédominance de la localisation olfactive dans 26.5% suivie de la convexité dans 23.5%. Nous n'avons rapporté aucune localisation intraventriculaire. Cette localisation représentait 11% des cas du méningiome chez la population pédiatrique [12] et 13,3% du méningiome de tout âge [18]. Contrairement à Stephane et coll [17], qui affirment que la plupart des méningiomes associés à un œdème cérébral sont de type II ou III, nous constatons que 2/3 des méningiomes avec un œdème perilésionnel sont de type I. Vivier et coll [19] en Afrique du sud ont rapporté une prédominance du type I estimée à 86,8%. Le méningiome atypique et malin représente 2% au cours de cette étude comme retrouvé par Wiemels et coll [20] pour qui le méningiome malin et atypique représente moins de 5% de l'ensemble des méningiomes. Pour les 3 patients ayant présenté des méningiomes extériorisés nous pensons que la consultation s’était faite à un stade trop avancé ce qui a permis d'observer ces formes évoluées. Comme l'ont rapporté Badiane et coll [1], le méningiome est primitivement intracrânien et s'est extériorisé secondairement par un processus lytique de la voute.

Tous les patients de notre série ont bénéficié d'un traitement chirurgical qui a consisté à la réalisation d'un volet puis d'une exérèse microchirurgicale par résection intra-capsulaire (debulking). Cette exérèse est appréciée selon le grade Sympson, et dépend essentiellement de la localisation. Elle est faite sans embolisation préalable, technique non disponible dans notre pays. Pour les méningiomes chirurgicalement inaccessible, selon Daniella [3] le traitement à base d'inhibiteur d'acide gras synthétase (cerulenin) décroitrait considérablement les cellules méningiales in vitro du fait d'une expression accrue d'acide gras synthétase chez 70% de méningiome atypique grade II et anaplasique. Nous avons recensé 6% de repousse tumorale entre 12 et 18 mois. L'exérèse tumorale chez ces patients était de sympson II. Ils ont été réopérés. Le méningiome associé à la neurofibromatose de type II est le plus souvent multiple, et sa présence est le marqueur de sévérité de cette pathologie [17]. Le décès des patients porteurs de méningiomatose est relatif à la délicatesse de la chirurgie de la base du crane car localisation qui constitue une difficulté opératoire. Les deux patients porteurs de la neurofibromatose sont décédés. Cela est corroboré par Baser et coll [21] selon lesquels le risque de mortalité est 2,5 fois supérieur en cas de neurofibromatose. Tous nos patients qui avaient consulté suffisamment tôt, ou qui avaient un méningiome de topographie aisément accessible avaient eu un bon résultat (38%). Les patients ayant été vus tard (stade de déficit neurologique; extériorisation de la tumeur) et ayant une localisation au niveau de la base du crane ont eu un résultat moins satisfaisant. La mortalité est de 16% au cours de cette étude. Elle est encore trop élevée pour une tumeur bénigne, mais tributaire de la topographie. La mortalité était de 12,63% en 2008 en Côte d'Ivoire [6]. Selon Hanna [22] la qualité de survie neurologique à long terme est détériorée chez les patients de plus de 45 ans opérés d'un méningiome de type I, ceci n'a pas pu être confirmé lors de cette étude.

## Conclusion

En dépit des avancées technologiques réalisées dans le monde, dans les domaines de diagnostic et de la prise en charge des méningiomes intracrâniens, les pays d'Afrique subsaharienne continuent d'accuser un retard certain malgré les efforts réalisés dans ce sens. Leur pronostic s'est considérablement amélioré à mesure de l'amélioration du plateau technique dans notre pays.
